# Novel Drift Reduction Methods in Foot-Mounted PDR System

**DOI:** 10.3390/s19183962

**Published:** 2019-09-13

**Authors:** Wenchao Zhang, Dongyan Wei, Hong Yuan

**Affiliations:** 1 University of Chinese Academy of Sciences, Beijing 100094, China; 2 Aerospace Information Research Institute, Chinese Academy of Sciences, Beijing 100094, China; yuanhong@aircas.ac.cn

**Keywords:** foot-mounted PDR, ZUPT-aided EKF, initial heading calibration, stationary phase detection, range constraint

## Abstract

The zero-velocity update (ZUPT)-aided extended Kalman filter (EKF) is commonly used in the traditional inertial navigation system (INS)-based foot-mounted pedestrian dead reckoning (PDR) system, which can effectively suppress the error growth of the inertial-based pedestrian navigation systems. However, in the realistic test, the system still often suffers from drift, which is commonly caused by two reasons: failed detection of the stationary phase in the dynamic pedestrian gait and heading drift, which is a poorly observable variable of the ZUPT method. In this paper, firstly, in order to improve the initial heading alignment accuracy, a novel method to calibrate the PDR system’s initial absolute heading is proposed which is based on the geometric method. By using a calibration line rather than only using the heading of the starting point, the method can calibrate the initial heading of the PDR system more accurately. Secondly, for the problem of failed detection of the stationary phase in the dynamic pedestrian gait, a novel stationary phase detection method is proposed, which is based on foot motion periodicity rather than the threshold comparison principle in the traditional method. In an experiment, we found that the zero-speed state points always occur around the minimum value of the stationary detector in each gait cycle. By taking the minimum value in each gait cycle as the zero-speed state point, it can effectively reduce the failed detection of the zero-speed interval. At last, in order to reduce the heading drifts during walking over time, a new motion constraint method is exploited based on the range constraint principle. During pedestrian walking, the distance between the foot position estimates of the current moment and the previous stationary period is within the maximum stride length. Once the distance is greater than the maximum stride length, the constraint method is used to confine the current estimated foot position to the sphere of the maximum stride length relative to the previous stationary foot position. Finally, the effectiveness of all proposed methods is verified by the experiments.

## 1. Introduction

The global navigation satellite system (GNSS) is a basic pedestrian positioning method. However, in urban canyons and indoor environments, GNSS positioning systems cannot be used due to signal attenuation and interference. The indoor pedestrian positioning system is a good complement to the GNSS positioning system. Many scholars proposed different methods for indoor pedestrian positioning systems, which mainly can be classified into two categories: infrastructure-based and infrastructure-less systems. Infrastructure-based positioning systems mainly use radio-frequency identification (RFID) [1], ultra-wide band (UWB) [2], Wi-Fi [3], orthogonal frequency division multiplexing (OFDM) signal [4,5], etc. These methods usually require pre-installation of the infrastructures in a given environment, which is costly and has limited application range. However, there are many kinds of applications which are hard to pre-install in certain infrastructures, e.g., in dangerous or collapsed buildings, emergency situations, etc. The infrastructure-less systems mainly use inertial measurement units (IMUs), barometers, pressure sensors, etc. These methods do not need to be pre-installed in any infrastructure, and can be widely used in an unknown environment. Among all the methods, the foot-mounted IMU has a wide range of applications, due to its independence from pre-installed infrastructures [6], and can be used to independently implement pedestrian positioning.

Since the IMU cannot obtain the geographic heading independently, initial heading calibration is necessary for the foot-mounted IMU system. As the IMU always suffers from drift, the position, velocity, and heading errors of the foot-mounted IMU grow with time. The zero-velocity updates (ZUPT) is a commonly used method to constrain the divergence of the inertial recursive positioning result. It assumes that, during walking, the foot touches the ground and remains stationary for a short time (stance phase) [7]. However, when pedestrians undergo various movements, most ZUPT methods usually cannot effectively detect the stationary period. Meanwhile, the heading error during pedestrian movement is unobservable for the ZUPT method [8]; thus, the ZUPT-assisted extended Kalman filter (EKF) algorithm is less restrictive for the heading, which means the heading will still diverge with time.

In pedestrian navigation systems, the initial heading is vital for the inertial based PDR system. If the initial heading cannot be effectively calibrated, the whole pedestrian positioning trajectory will be offset from the true trajectory. At present, most methods only use the information of the starting point (including geographic direction, geomagnetic heading, etc.) to achieve initial alignment of the PDR system. However, the direction of the pedestrian’s foot usually does not exactly coincide with the geographic heading of the starting point. Moreover, the geomagnetic heading is always subjected to interference, especially when only using the starting point’s geomagnetic heading. For example, Nilsson et al. [9] proposed an IMU initial alignment algorithm based on accelerometers and magnetometers. Nevertheless, local magnetic distortions may still corrupt the estimated heading, and the alignment accuracy can be further decreased by magnetic interference. Xing et al. [10] proposed the rotation modulation technique at the initial stage, which is an option for initial alignment of the IMU; however, a rotating platform is needed, and the initial alignment time is too lengthy.

The detection of the stance phase is a key step of the ZUPT method, and many detection methods were developed [11,12,13,14]. Commonly used detection methods include the acceleration magnitude method [15], the angular velocity magnitude method [16,17,18,19], the moving variance method [20], or a combination of the above methods [21,22,23]. In addition, there are some other methods, such as, in Reference [14], where the zero-speed interval was determined based on a likelihood ratio test (LRT) detector. The detector provides good performance at low gait speeds (approximately 0.83 m/s). In Reference [11], a segmentation based on the gyro output was used to construct a hidden Markov model-based algorithm. The algorithm exhibited good reliability under walking and running conditions. However, the state transition model was complex and difficult to implement. In Reference [12], a standing phase detector consisting of a foot detector and two zero-speed detectors was proposed. The detector could successfully detect zero-speed during walking, climbing stairs, and running. However, when pedestrians alternated between walking and running, the detectors were easily confused. Most of these methods above have a common characteristic, whereby the stationary phase detection is based on setting a threshold, by comparing the detector with the threshold to determine whether the current moment is in the stationary phase or in the dynamic pedestrian gait. These methods have a significant advantage when the pedestrian exhibits a single motion, which means the magnitude of the stationary phase detector is basically kept within a stable range. However, in the case of movement including walking, running, going upstairs and downstairs, etc., the zero-speed detection threshold fluctuates greatly with the movement condition, and there cannot be a universally applicable threshold that can effectively detect the stationary phase under various pedestrian movements.

As pointed out in Reference [8], another main drawback of the existing foot-mounted PDR system is the systematic heading drift. The ZUPT-aided EKF algorithm is less restrictive for the heading. The estimated trajectories drift away from the actual path as time progresses. Hence, many scholars conducted in-depth studies. Zero-angular rate update (ZARU) is a technique that can help to estimate the bias of the gyroscope in every still phase [21]. However, during the stance phases, there are systematic motions and residual angular rates that exist while walking [8]. The ZARU normally does not really happen, except when the user/pedestrian has a stable stance position for a long period of time. False ZARU can harm the heading estimation of the system severely [24]. Jiménez et al. [21] proposed a technique called heuristic drift reduction (HDR) to reduce the heading error, based on the fact that most walls and corridors inside buildings are made up of straight lines, using only four or eight dominant directions as the reference directions. The HDR algorithm is suitable for pedestrians walking along the dominant directions; however, sometimes, pedestrians do not always walk along the dominant directions. Hong et al. [25] adopted a geomagnetic correction algorithm to correct the heading angle by taking the difference of courses computed by the magnetometer and attitude matrix as a measurement. Afzal [26] estimated the heading error by capturing changes in the magnetic field measured by magnetometers in the pedestrian’s stationary state. Since the magnetometer suffers more perturbations in indoor environments, the reliability of its computed heading angle cannot be guaranteed. Skog [27] proposed that, during the dynamic pedestrian gait, another important observation to be made is that the drifts obtained are symmetrical. These errors are large-scale manifestations of modeling errors in the system. One possible way these errors can be mitigated is to use a foot-mounted inertial navigation system (INS) on both feet, since there is limit on the separation between the two feet equipped with a ZUPT-aided INS. Thus, the symmetrical modeling heading errors can be canceled out.

Based on the analysis of the current foot-mounted PDR system, in order to improve its robustness, in this paper, we make three contributions.

Firstly, in order to constrain the initial heading error, a new method to calibrate the PDR system’s initial absolute heading is proposed, which is based on geometric methods. The purpose of the initial calibration is to rotate the test trajectory with the actual one. Since the plane trajectory is two-dimensional, a more straightforward method is to directly align a part of the trajectory with its actual trajectory at the initial stage; then, the entire trajectory can be calibrated. The principle of the foot-mounted IMU system is that the increment (including position increment, velocity increment, and attitude increment) is obtained by the IMU measurement. Then, the navigation information can be calculated by the sum of the initial value and the increment. It is assumed that the pedestrian’s initial state is standing at one point; thus, the initial velocity value is zero. The initial position can be obtained by GNSS or set to zero. The difficulty for a foot-mounted IMU system is the determining the initial heading. In this paper, at the initial stage, the heading of the initial point is set to 0; after that, the pedestrian walks along a known line. Then, the test trajectory of this line is obtained by the foot-mounted IMU system, directly rotating the test track to coincide with the known line, i.e., the calibration of the entire trajectory is completed. To calibrate the PDR system, we used the difference between the estimated heading and true heading of the known line.

Secondly, a novel stationary phase detection method is proposed to improve the effect of the stationary phase detection in the dynamic pedestrian gait. The current zero-speed detection method is mainly based on the principle of threshold comparison. The main drawback is that the zero-speed detection threshold fluctuates greatly with the movement condition, and there is no universally applicable threshold that can effectively detect the stationary phase under various pedestrian movements. In an experiment, we found that the zero-speed points in each gait cycle always occur around the minimum value of the zero-speed interval detector. Base on that rule, we use the periodic gait-cycle window to divide the pedestrian movement into discrete gait cycles; then, we take the minimum value in each gait cycle as the zero-speed state point. Compared to the existing methods, the proposed method does not need to set the zero-speed detection threshold, and performs well for zero-speed interval detection under various pedestrian movements.

Thirdly, despite having an initial heading calibration phase, the heading drift is a poorly observable variable for the ZUPT-aided PDR method; thus, the systematic heading still drifts over time. In order to reduce the heading drift during pedestrian movement, we exploit a new motion constraint method based on the range constraint principle. During the pedestrian movement, the distance between the foot position estimates of the current moment and the previous stance instance is within the maximum stride length. Once the distance is greater than the maximum stride length, the constraint method is used to confine the current estimated foot position to the sphere of the maximum stride length relative to the previous stance foot position.

The layout of this paper is as follows: in Section 2, we describe the new method to calibrate the PDR system’s initial absolute heading. Section 3 explains the novel stationary phase detection method. In Section 4, we present the use of the proposed range constraint method to suppress the heading drifts during the pedestrian movement. In Section 5, the three proposed methods in this paper are analyzed in detail, and compared with the existing methods, where the effectiveness of the proposed methods is shown. At last, in Section 6, we conclude this paper’s work and offer some future research suggestions.

## 2. Using a Calibration Line to Calibrate the PDR System’s Initial Heading

**Figure 1:**
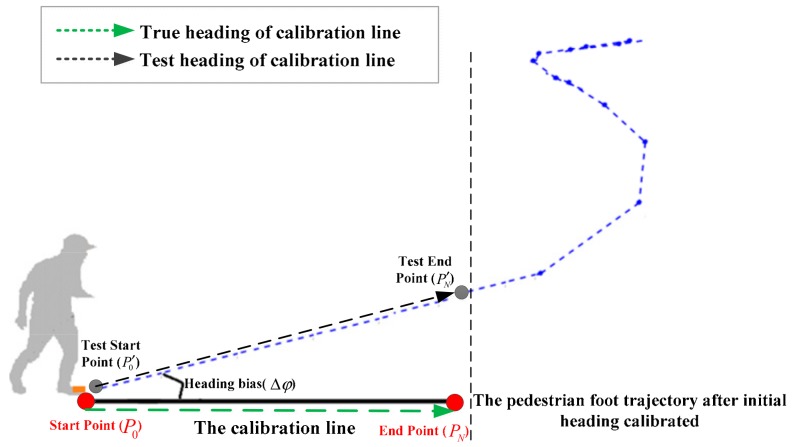
The method to calibrate the pedestrian dead reckoning (PDR) system’s initial absolute heading.

The proposed method to calibrate the PDR system’s initial absolute heading is based on the geometric method which mainly involves using a calibration line rather than the heading of the starting point. As shown in Figure 1, the coordinates of the start and end points of the calibration line are already known (obtained by DGNSS (differential global navigation satellite system), whose positioning accuracy is within a centimeter). Therefore, the true heading of the calibration line can be obtained, which can be denoted as $\varphi_{true}$. In order to calibrate the initial heading of the inertial-based PDR system, the pedestrian needs to walk along the calibration line (the length of the calibration line is generally 5–10 m) at the beginning; then, the test coordinates of the start and end points of the calibration line can be obtained using the PDR system. Using the test coordinate difference of the start and end points of the line, the estimated heading of the line can be obtained, which can be denoted as $\varphi_{test}$. Hence, the initial heading bias of the PDR system relative to the true heading (the heading of the calibration line) is $\Delta \varphi =\varphi_{true}-\varphi_{test}$. With the heading bias, the initial absolute heading of the PDR system can be calibrated correctly.

The method to calibrate the PDR system’s initial absolute heading is shown in Algorithm 1.


**Algorithm 1: The Algorithm to Calibrate the PDR System’s Initial Absolute Heading**
**Initiate:** at time t←0
$\varphi_{0}\leftarrow 0$: the initial heading of the inertial-based foot-mounted PDR system $P_{0}{}^{\prime }\leftarrow (x_{0},y_{0},z_{0})$: the initial position of the inertial-based foot-mounted PDR system 
**Input:**
$P_{0}$ and $P_{N}$: the start and end points of the calibration line obtained by DGNSS 
$\varphi_{true}$: the absolute heading of the calibration line computed from the coordinate difference of $P_{0}$ and $P_{N}$
**Output:**Δφ: the initial heading bias of the inertial-based foot-mounted PDR system**Step 1:** Get the test coordinates of the start and end points of the calibration line  When the pedestrian (aided by the inertial-based PDR system) walks along the calibration line, all the coordinates of the line can be obtained, including the start point $P_{0}{}^{\prime }$ and end point $P_{N}{}^{\prime }$. 
**Step 2:** Get the test heading of the calibration line ($\varphi_{test}$)  Using the test coordinates of the start and end points, the test heading $\varphi_{test}$ of the calibration line can be obtained. The computing equation is shown in Equation (1). **Step 3:** Get the initial heading bias of the foot-mounted PDR system ($\Delta \varphi =\varphi_{true}-\varphi_{test}$)  Using $\varphi_{test}$ and $\varphi_{true}$ of the calibration line, the initial heading bias of the PDR system can be computed: $\Delta \varphi =\varphi_{true}-\varphi_{test}$.

**Figure 2:**
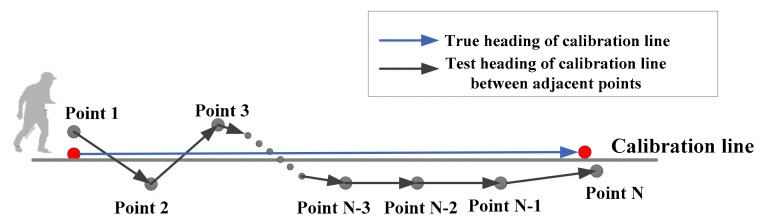
The geometric method to calibrate the PDR system’s heading by averaging the heading of adjacent points obtained by the PDR system.

Another method to calibrate the initial heading of PDR system is using the average test heading of the calibration line. The method described above only uses the test coordinates of start and end points of the calibration line. However, when the pedestrian moves along the calibration line, all the test coordinates of the line (each time the foot touches the ground) can be obtained by the ZUPT-aided PDR system. Thus, the heading between each pair of adjacent points can be computed using Equation (1). Then, averaging all the headings of the adjacent points, the test heading of the line can also be obtained, as shown in Figure 2.

In Figure 2, the true heading of the calibration line can be computed from the coordinate difference of the start and end points (red points in Figure 2, which are obtained by DGNSS). Point 1, Point 2, …, Point N are all the test points obtained by the PDR system when the pedestrian moves along the calibration line. The heading between each pair of adjacent points can be obtained using Equation (1) as follows:

(1) $$\varphi =\left\{\begin{array}{cc} -\frac{\pi }{2} & \left(\Delta x=0,\Delta y<0\right)\\ \frac{\pi }{2} & \left(\Delta x=0,\Delta y>0\right)\\ \pi +\mathrm{atan}2(\Delta y/\Delta x) & \left(\Delta x<0,\Delta y\geq 0\right)\\ -\pi +\mathrm{atan}2(\Delta y/\Delta x) & \left(\Delta x<0,\Delta y<0\right)\\ \mathrm{atan}2(\Delta y/\Delta x) & \left(\Delta x>0\right) \end{array}\right.$$

where Δx and Δy are the coordinate differences of the adjacent points. The range of the heading is within (−π,π). Then, the average heading of the adjacent points can be obtained as follows:

(2) $$\varphi_{ave}=\frac{1}{N-1}\sum_{i=1}^{i=N-1}\varphi_{i}$$

where *N* is the number of the points, and $\varphi_{i}$ is the heading of adjacent points. Hence, the test heading of the calibration line is $\varphi_{test}=\varphi_{ave}$. In theory, $\varphi_{ave}$ may average some deviation headings (see Figure 2, the headings of Point 1 to Point 2 and Point 2 to Point 3 deviate from the actual walking direction). Thus, the heading $\varphi_{ave}$ is less realistic in representing the heading of the calibration line.

By comparing the test heading of the calibration line with the true heading, the initial heading bias of the PDR system can be computed as follows:

(3) $$\Delta \varphi =\varphi_{true}-\varphi_{test}$$

Using the heading bias Δφ, the initial absolute heading of the PDR system can be calibrated correctly. The proposed two methods above are compared with two existing methods: (1) directly using the absolute geographical heading of the start point to initialize the heading of the PDR system, and (2) using the geomagnetic field to initialize the PDR system. The effectiveness of the proposed method is shown in the experimental evaluation section of the paper.

## 3. The Novel Stationary Phase Detection Method

The use of IMU sensors to obtain high precision of human motion positioning is challenging because it is largely affected by the drift of the IMU sensors. Fortunately, the human foot gait includes two stages: standing and swinging [28]. This can be used to estimate the impact of IMU drift. In the standing stage, human feet stand at the ground; therefore, their actual speed is close to zero. If the IMU’s foot speed at this stage is different from zero, it must be due to an error caused by the IMU drift. Then, we can apply the ZUPT algorithm to reduce the drift of the IMU and improve the accuracy of the positioning. Therefore, the accuracy of the standing phase detection is crucial for achieving higher human foot positioning accuracy.

### 3.1. Gait Characteristics Analysis

**Figure 3:**
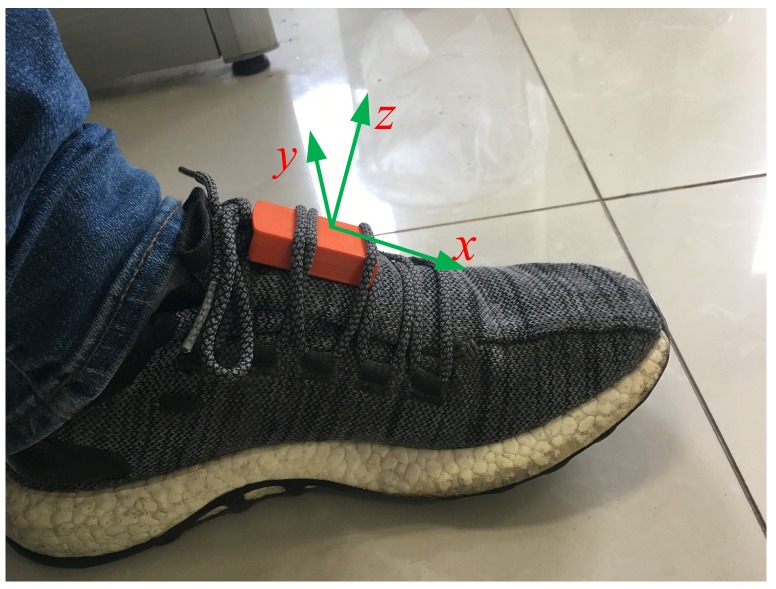
The inertial navigation system (INS)-based foot-mounted PDR system.

The pedestrian navigation shoe based on a self-contained sensor is shown in Figure 3, where all of the sensors are integrated in a structure to constitute an IMU, which includes a three-axis accelerometer and three-axis gyroscope. The IMU is fixed on the foot surface. When the pedestrian starts walking, the IMU constantly measures the acceleration and the angular rate of foot motion.

**Figure 4:**
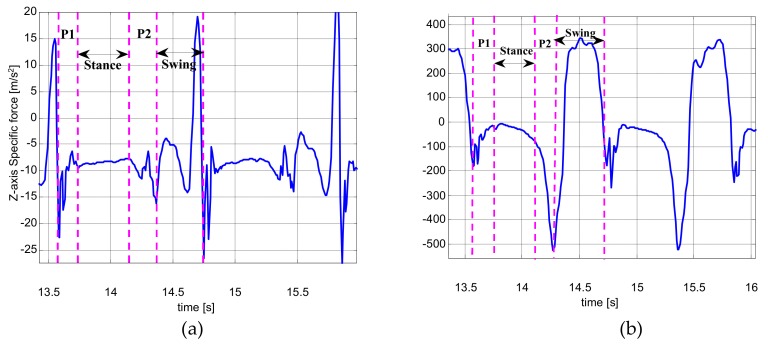
Pedestrian gait cycles: (**a**) two gait-cycle outputs of *z*-axis accelerometer; (**b**) two gait-cycle outputs of *y*-axis gyroscope.

The pedestrian gait cycle shown in Figure 4 is obtained using the navigation shoe to collect the inertial parameters of a pedestrian’s foot motion during movement. Figure 4a stands for the *z*-axis accelerometer output, which denotes the most varied acceleration, and Figure 4b represents the *y*-axis gyroscope output, which is the dominant rotation axis during movement. Figure 4a,b have two pedestrian gait cycles of the same period. The first gait cycle is divided into four stages, which are P1, stance, P2, and swing. P1 stage stands for the process from the heel striking the ground to the front sole striking the ground. Stance stage is the front sole contacting the ground completely, during which the sensors’ outputs are approximately constant. The *y*-axis gyroscope output is not exactly zero, as there are systematic motions and residual angular rates existing [8]. The accelerometer output is approximately the gravitational acceleration. Due to the way the IMU is mounted (see Figure 3), the *z*-axis of the IMU does not exactly coincide with the gravity axis; thus, the component of the *z*-axis is not exactly equal to gravitational acceleration. This period is also called the zero-velocity interval, which is the most important stage, by detecting the periodic zero-velocity interval during pedestrian movement, the ZUPT algorithm can effectively reduce the drift of the IMU and improve the accuracy of the navigation shoe’s positioning. After the stance stage, the lift foot stage (P2) starts from the heel of the foot lifting off the ground to the moment of the toe. After that, the foot lifts off the ground, the leg begins to swing, and the body moves forward, which is the swing stage. After the swing phase, the heel of the foot strikes the ground again, which marks the beginning of another gait cycle.

### 3.2. Analysis of the Existing Stationary Phase Detection Method

**Figure 5:**
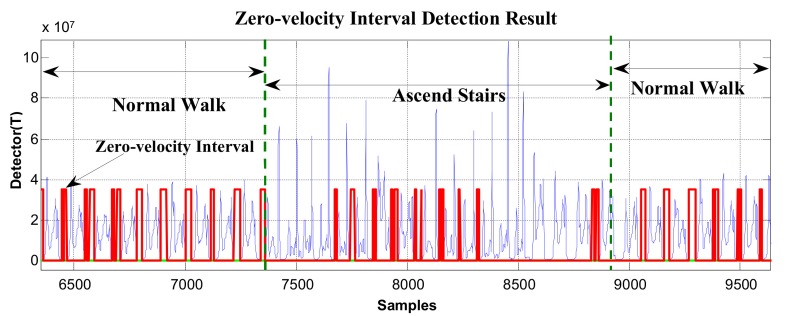
The zero-velocity interval detection result containing both normal walking and ascending stairs.

**Figure 6:**
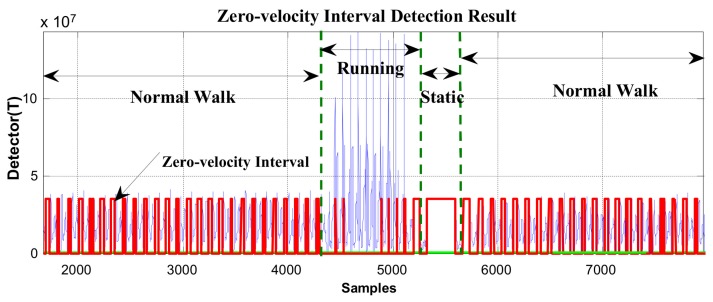
The zero-velocity interval detection result containing both normal walking and running.

The majority of zero-velocity detection methods employ comparisons between thresholds and the magnitude of acceleration, moving variance of acceleration, magnitude of angular rate, or their combinations. The primary limitation of these methods is that the variations in acceleration and angular rate differ greatly under various movement modes, such as walking, running, stair-climbing, etc. Thus, it is difficult to find a threshold function or threshold value that is widely applicable. We demonstrate this in Figure 5 and Figure 6.

The general likelihood ratio test (GLRT) method [14], which is the most commonly used method to detect the zero-velocity interval, is employed in Figure 5 and Figure 6. It uses the output of both the accelerometers and gyroscopes during the pedestrian movement. Therefore, its zero-velocity interval detection result is better than other algorithms based on threshold comparison. By using this method, the constructed zero-velocity interval detector *T* can be denoted as follows:

(4) $$T\left(z_{n}^{a},z_{n}^{w}\right)=\frac{1}{W}\sum_{k=n}^{n+W-1}\left(\frac{1}{\sigma_{a}^{2}}{\left\|y_{k}^{a}-g\frac{{\bar{y}}_{n}^{a}}{\left\|{\bar{y}}_{n}^{a}\right\|}\right\|}^{2}+\frac{1}{\sigma_{w}^{2}}{\left\|y_{k}^{w}\right\|}^{2}\right)$$

where $y_{k}^{a}$ and $y_{k}^{w}$ denote the specific force vector and angular rate vector, respectively. $\sigma_{a}^{2}$ and $\sigma_{w}^{2}$ denote the variance of the measurement noise of the accelerometers and gyroscopes, respectively. Furthermore, ${\left\|a\right\|}^{2}=a^{T}a$, where ${\left(\cdot \right)}^{T}$ denotes the transpose operator. Moreover, ${\bar{y}}_{n}^{a}$ denotes the sample mean, i.e.,

(5) $${\bar{y}}_{n}^{a}=\frac{1}{W}\sum_{k=n}^{n+W-1}y_{k}^{a}$$

The principle of the GLRT method is that, by comparing the magnitudes of detector *T* in Equation (4) with a threshold, it can determine whether the current moment is in the zero-velocity interval.

In Figure 5 and Figure 6, the red line represents the stationary state and moving state, where large values indicate the zero-velocity points and small values indicate the moving state. Figure 5 shows the zero-velocity interval detection result of movement containing both normal walking and ascending stairs. As we can see, although the threshold of the GLRT detector performs well during normal walking, when the pedestrian ascends stairs, the threshold fails to detect the zero-velocity interval during this period (near Sample 7500 and Sample 8500 in Figure 5). Figure 6 shows the zero-velocity interval detection result of movement containing both normal walking and running. Similarly, the threshold of GLRT detector performs well during normal walking, while it fails during the running period (between Sample 4000 and Sample 5000 in Figure 6).

In addition, in the literature [29,30], some adaptive threshold zero-velocity interval detection algorithms were proposed. The basic detection principle is the same as the GLRT method, which mainly involves constructing the zero-speed interval detector and comparing the detector with the threshold value to determine whether the current moment is in the zero-velocity interval. However, these algorithms can adaptively change the detection threshold using the established approximation relationship between the motion characteristics and the threshold. However, the effect of this method is not obvious for the random motion generated during actual movement. At the same time, the adaptive model algorithm is not universally applicable to different people.

### 3.3. Novel Stationary Phase Detection Method Based on Foot Motion Periodicity

In Section 3.1 we pointed out that pedestrian footsteps are periodically moving; by detecting the periodic zero-velocity interval during pedestrian movement, the ZUPT algorithm can effectively reduce the drift of the IMU and improve the accuracy of the navigation shoe’s positioning. However, as shown in Section 3.2 the primary limitation of the existing stationary phase detection method is that the variations in acceleration and angular rate differ greatly under different motion modes, such as walking, running, stair-climbing, etc. Thus, it is difficult to find a threshold function or threshold value that is widely applicable. In this section, a novel stationary phase detection method is proposed, which is not based on the threshold comparison principle but based on foot motion periodicity.

**Figure 7:**
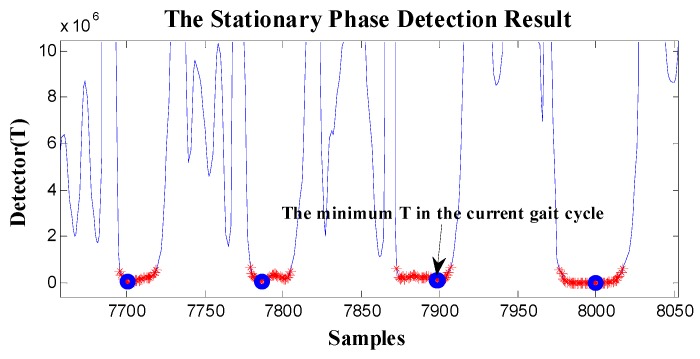
The stationary phase detection result during foot movement. Red asterisks denote the detection results of the general likelihood ratio test (GLRT) method, and the blue circles denote the minimum value of detector *T* in each gait cycle.

At first, the zero-velocity interval detector *T* should be constructed. As Equation (4) is a universally useful method, which uses the output of both accelerometers and gyroscopes during foot motion, it is still used to construct the zero-velocity interval detector in our method. By analyzing the zero-speed detection results of Figure 5 and Figure 6, it was found that the zero-speed state points in each gait cycle always occurred around the minimum value of the detector *T*. As shown in Figure 7, the red asterisks denote the detection results of the GLRT method, and the blue circles denote the minimum detector point in each gait cycle. The red asterisks are always close to the blue circles, which is a universally applicable rule to all kinds of movement, including walking, running, stair-climbing, etc. Therefore, if the zero-speed state points are to be detected accurately in each gait cycle under various motion states, the key is to find the minimum value of the detector *T* in the period, before simultaneously selecting some neighboring points as the zero-speed points.

Similar to the principle of the global positioning system (GPS)/INS combined navigation system, for the ZUPT-aided PDR system, we only need to periodically correct the inertial recursive result; then, the divergence of the inertial recursive position results can be constrained. There is no need to detect all the zero-speed state points in each gait cycle, and it is only necessary to ensure that the detected zero-speed state points are all correct in each gait cycle. The proposed method can ensure just that.

Actually, in the experimental evaluation section of the paper, we prove that, in each gait cycle, as long as one accurate zero-speed point (the minimum value point of detector *T*) is detected correctly, the ZUPT-aided PDR algorithm can effectively suppress the positioning errors of the inertial recursive results. Also, the proposed method is compared with the GLRT method to verify its effectiveness.

Secondly, in order to correctly find the minimum value of detector *T* in each cycle, the pedestrian movement should be divided effectively according to the gait-cycle duration. However, during the actual pedestrian motion, there are various motion states, such as walking, fast walking, running, slow running, fast running, stair-climbing, going downstairs, etc. Thus, the time length of the gait cycle is different under different motions. Therefore, a suitable gait-cycle duration should be chosen to adapt to various pedestrian motions. As analyzed in the literature [31], for an adult walking at normal speed, the cycle time is within 1 to 1.2 s. In our experiments, we found that, regardless of the type of motion (including walking, running, stair-climbing, going downstairs, etc.), generally, at least one gait cycle can be completed within 0.7–1.2 s. If the gait cycle duration is longer than 1.2 s during normal movement, it must because the pedestrian has a pause or is in a standstill state during the current gait cycle. Therefore, in that case, a static detection algorithm is added to the proposed method to detect the pause or standstill state during the gait cycle. If the standstill or pause state is detected, the previous navigation state (position, velocity, and attitude) is used as the current navigation state to constrain the divergence of the inertial recursive results.

**Figure 8:**
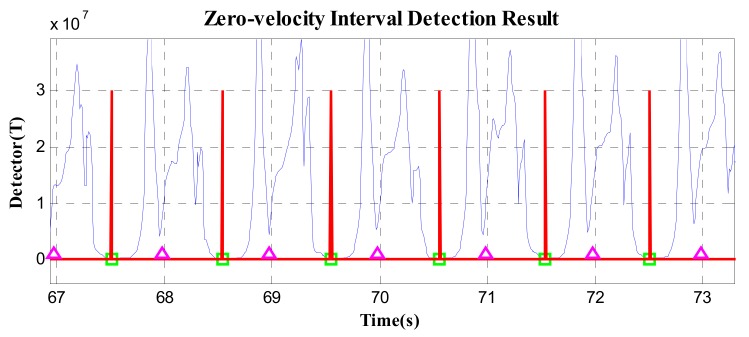
The pedestrian movement result divided according to the cycle duration.

According to the proposed method, in Figure 8, the pedestrian movement is effectively divided into independent gait cycles. In the figure, the magenta triangle is the time instant that indicates an equal time interval (gait cycle). The green square is the minimum value of detector *T* in each equal time interval, that is, the zero-speed state point.

At last, in each gait cycle, by comparing the value of all the detectors, the minimum value point can be effectively found, which is the zero-speed point of the current gait cycle.

**Figure 9:**
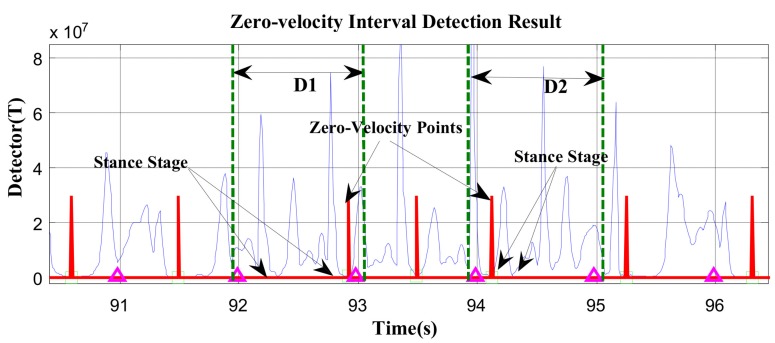
In D1 and D2, there are two stance stages, but only one zero-speed point.

**Figure 10:**
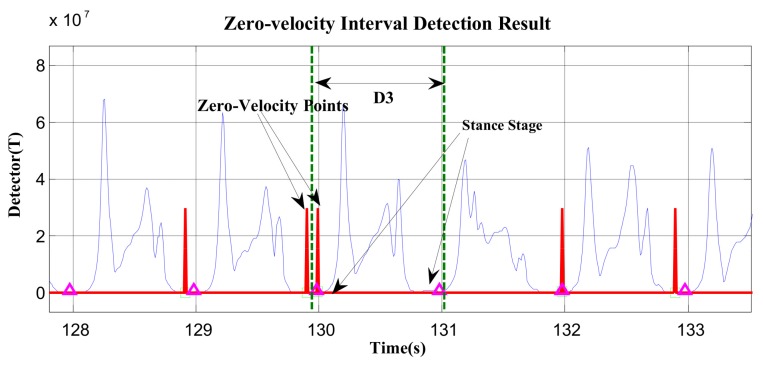
In D3, the zero-speed point is missed in the current stance stage (at 131 s).

In addition, the proposed zero-speed point detection method may fail in two cases. Firstly, for fast walking, running, or other short gait-cycle movement, there may be 2–3 gait cycles in the 0.7–1.2-s cycle duration. Thus, the detected zero-speed point is the common minimum *T* point of the current 2–3 gait cycles; in other words, there are 1–2 gait cycles that missed detection. As shown in Figure 9, in D1 and D2, there are two stance stages in the current cycle duration, but only one zero-speed point (the common minimum *T* point) of the two stance stages was detected. Secondly, as shown in Figure 10, the current cycle duration D3 starts from the previous stance stage and ends at the current stance stage. The detector *T* in the previous stance stage is smaller than that in the current stage; thus, the zero-speed point is still detected in the previous stance stage, rather than in the current stage.

Although missed detection occurs in the above two cases, the proposed method can ensure that at least one correct zero-speed point is effectively detected within the set 0.7–1.2-s cycle duration, and there is no false detection. That is to say, the algorithm effectively corrects the inertial recursive position result every 0.7–1.2 s.

**Figure 11:**
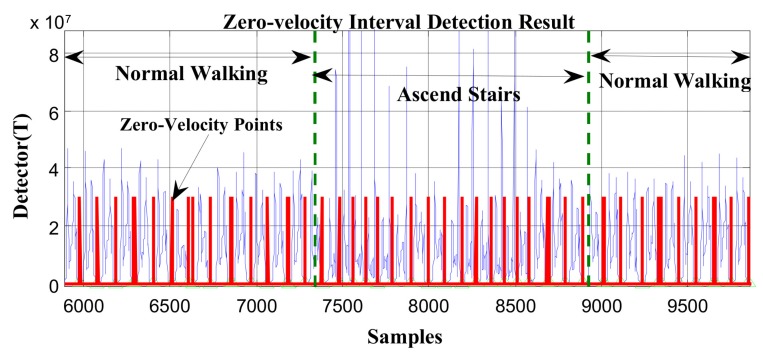
The zero-velocity point detection result of movement containing both normal walking and ascending stairs using the proposed method.

**Figure 12:**
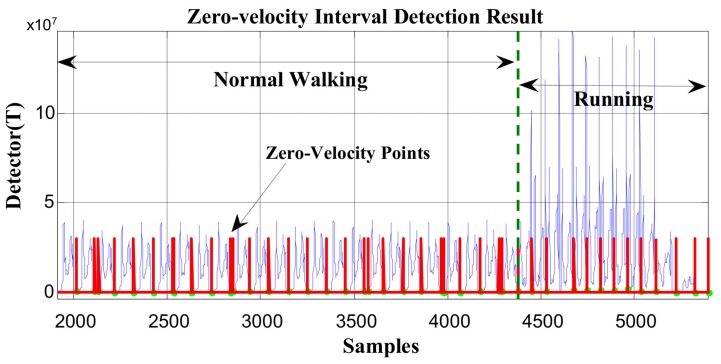
The zero-velocity point detection result of movement containing both normal walking and running using the proposed method.

In order to verify the effectiveness of the proposed method, the data in Figure 5 and Figure 6 were reprocessed using the proposed method, and the results are shown in Figure 11 and Figure 12. Compared with the case of missed detection near Sample 7500 and Sample 8500 after switching from normal walking to ascending stairs in Figure 5, it can be seen from Figure 11 that the missed detection situation is effectively improved. At the same time, compared to the case of missed detection between Sample 4000 and Sample 5000 after switching from normal walking to running in Figure 6, in Figure 12, although there is still one gait cycle missed for the minimum *T* point, the overall zero-speed point detection is improved.

The proposed method to detect the zero-speed points under various movements is described in Algorithm 2.


**Algorithm 2: The novel stationary phase detection algorithm**
**Initiate:** at time t←0
*Threshold_0_*: Only using the static detection algorithm *t*: The periodic time window to divide the pedestrian movement into independent gait cycles**Input:***T*: The stationary phase detector constructed using Equation (4), which uses the output of both accelerometers and gyroscopes during foot motion**Output:***zupt*: The detection result of zero-speed points during dynamic gait cycles**Step 1:** Static detection  Use the static detection algorithm to determine whether the pedestrian is in a static state. If the pedestrian in a static state, directly use the previous navigation state (position, velocity, and attitude) as the current navigation state to constrain the divergence of the inertial recursive results. If in a dynamic phase, execute Step 2. **Step 2:** Zero-speed point detection using foot motion periodicity  Divide the detector variable *T* using the periodic window *t*, and, within the period window, the minimum value of the detector *T* is the zero-speed point. **Step 3:** Repeat Step 1 and Step 2 to continue detecting zero-speed points of the current time window *t* until the pedestrian ends the movement.

The existing zero-speed detection method is mainly based on the principle of threshold comparison. The drawback is that the detection effect is poor for various pedestrian motions. In this section, assuming the pedestrian movement is cyclical, a new zero-speed detection method is proposed. The method uses the pedestrian motion periodicity to divide the pedestrian motion according to a reasonable cycle length time, and takes the minimum value of the detector in the period as the zero-speed state point. The method does not need to set the zero-speed detection threshold, and performs well for zero-speed detection under various pedestrian movements.

## 4. The Maximum Stride-Length Constraint Algorithm

Using the algorithm proposed in Section 3, the zero-speed state points in the gait cycle can be effectively and accurately detected under different motion modes; then, the ZUPT algorithm can constrain the divergence of the positioning results. However, the heading error is unobservable for the ZUPT method [8]; thus, the ZUPT-assisted EKF algorithm is less restrictive for the heading. This means that, during walking or running, the pedestrian heading will still diverge over time, which will result in the estimated trajectories drifting away from the actual path as time progresses. Despite having a calibration phase (in Section 1) at the initial stage, systematic heading drifts are still persistent.

In this section, in order to reduce the systematic heading drift during the foot motion period, we exploit a new motion constraint method based on the range constraint principle [32,33]. The basic theory mainly refers to the content in Reference [33]. However, the method in Reference [33] is aimed at the range constraint of two feet, and the method we propose is for the range constraint of one foot. During pedestrian walking, the distance between the foot position estimates of the current time instance and the previous stationary instance is within the maximum stride length. Once the distance is greater than the maximum stride length, the proposed constraint method is used to confine the current estimated foot position to the sphere of the maximum stride length relative to the previous stationary foot position.

### 4.1. Foot Range Constraint Analysis

**Figure 13:**
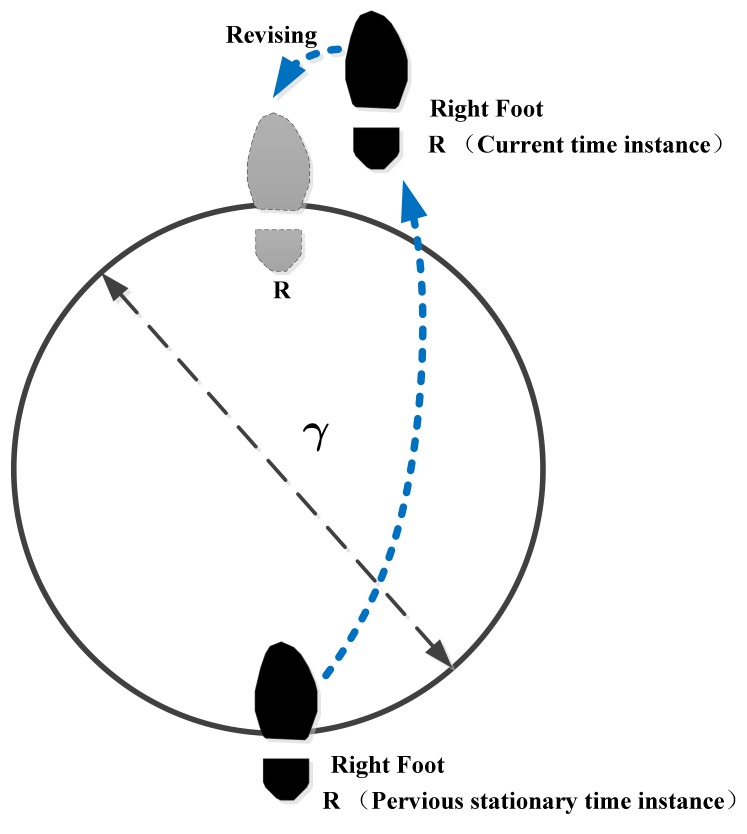
Relationship between current foot position and previous stationary foot position.

Usually, the step length is defined as the interval between two successive heel-strike events of alternate feet [31], while the stride length in the paper is the interval between two successive heel-strike events of the ipsilateral foot. As shown in Figure 13, the separation between the current foot position and the previous stationary period foot position is the stride length. Therefore, the stride length in the paper is twice that of the step length. By studying the foot movement, we notice that the separation of the foot position between the current time instance and the previous stationary period does not exceed a certain threshold value. In other words, when the foot position of the previous stationary period is already known, the distance of the current foot position relative to the previous stationary period cannot exceed a threshold value, which we define as the maximum stride-length bound. Let γ be the maximum stride length (see Figure 13). By referring to the analysis results in the literature [34,35], as stated in Reference [34], the step length is about 0.75 m for healthy adults walking at their self-selected speed. In Reference [35], the average step length varies for males and females: around 0.79 m for males and 0.66 m for females. Therefore, generally, the average maximum stride length is around 1.58 m for general adults.

Based on the above analysis, during pedestrian movement, when the foot is stationary, with the help of the proposed algorithm in Section 3, the error covariance can be minimized by the ZUPT algorithm. While the foot is in motion, by using the maximum stride-length bound method, the position error can be effectively suppressed.

Let $x_{k-1}^{Stationary}\in {\mathbb{R}}^{9}$ be the navigation state vector of the previous stationary period, defined as

(6) $${\hat{x}}_{k-1}^{Stationary}≜{\left[\begin{array}{ccc} {\hat{p}}_{k-1}^{S} & {\hat{v}}_{k-1}^{S} & {\hat{\theta }}_{k-1}^{S} \end{array}\right]}^{T}$$

where ${\hat{p}}_{k-1}^{S}\in {\mathbb{R}}^{3}$, ${\hat{v}}_{k-1}^{S}\in {\mathbb{R}}^{3}$, and ${\hat{\theta }}_{k-1}^{S}\in {\mathbb{R}}^{3}$ are the position, velocity, and attitude (roll, pitch, and yaw angles) estimates, respectively, of the previous stationary period by using the ZUPT-aided PDR method.

Let ${\hat{x}}_{k}\in {\mathbb{R}}^{9}$ be the navigation state vector of the current time instance, defined as

(7) $${\hat{x}}_{k}^{Current}≜{\left[\begin{array}{ccc} {\hat{p}}_{k}^{C} & {\hat{v}}_{k}^{C} & {\hat{\theta }}_{k}^{C} \end{array}\right]}^{T}$$

where ${\hat{p}}_{k}^{C}\in {\mathbb{R}}^{3}$, ${\hat{v}}_{k}^{C}\in {\mathbb{R}}^{3}$, and ${\hat{\theta }}_{k}^{C}\in {\mathbb{R}}^{3}$ are the position, velocity, and attitude (roll, pitch, and yaw angles) estimates, respectively, at the current time instant $k\in N^{+}$.

According to the derivation process of the range constraint algorithm for two feet in Reference [33], the range constraint algorithm for one foot is derived as shown below.

The vector combining the navigation state vector of the previous stationary period and the current time instance is defined as follows:

(8) $${\hat{p}}_{k}≜{\left[\begin{array}{cc} {\left({\hat{p}}_{k-1}^{S}\right)}^{T} & {\left({\hat{p}}_{k}^{C}\right)}^{T} \end{array}\right]}^{T}$$

The matrix is defined as follows:

(9) $$L≜\left[\begin{array}{cc} I_{3} & -I_{3} \end{array}\right]$$

where $I_{q}$ is the identity matrix of size q.

It is intuitive that, at any instant of time $k\in N^{+}$, the distance between the position estimates of the current instance and the previous stationary period cannot be greater than γ, i.e., the following condition must hold:

(10) $${\left\|L{\hat{p}}_{k}\right\|}_{2}={\left\|{\hat{p}}_{k-1}^{S}-{\hat{p}}_{k}^{C}\right\|}_{2}\leq \gamma ,\;\forall k\in {\mathbb{N}}^{+}$$

In the constrained least squares (CLS) framework, the range constraint problem is formulated as

(11) $$p_{k}=\underset{p\in {\mathbb{R}}^{6}}{\mathrm{argmin}}\left({\left\|{\hat{p}}_{k}-p\right\|}_{2}^{2}\right)\;s.t.\;{\left\|Lp_{k}\right\|}_{2}^{2}\leq \gamma^{2}$$

where $p_{k}$ is the solution of the constrained least squares problem, defined as

(12) $$p_{k}≜{\left[\begin{array}{cc} {\left(p_{k-1}^{S}\right)}^{T} & {\left(p_{k}^{C}\right)}^{T} \end{array}\right]}^{T}$$

The Lagrange function for Equation (11) with Lagrange multiplier λ is given as follows [36]:

(13) $$J\left(p_{k},\lambda \right)≜{\left\|{\hat{p}}_{k}-p_{k}\right\|}_{2}^{2}+\lambda \psi (p_{k})$$

where

(14) $$\psi (p_{k})={\left\|Lp_{k}\right\|}_{2}^{2}-\gamma^{2}$$

The Lagrangian condition yields

(15) $$\frac{\partial J\left(p_{k},\lambda \right)}{\partial p_{k}}=0\Rightarrow \left(I_{6}+\lambda L^{T}L\right)p_{k}={\hat{p}}_{k}$$

(16) $$\frac{\partial J\left(p_{k},\lambda \right)}{\partial \lambda }=0\Rightarrow \psi (p_{k})=0$$

The solution of Equation (15) is

(17) $$p_{k}={\left(I_{6}+\lambda L^{T}L\right)}^{-1}{\hat{p}}_{k}$$

Solving for λ, firstly note that

(18) $${\left(I_{6}+\lambda L^{T}L\right)}^{-1}=\frac{1}{1+2\lambda }\left[\begin{array}{cc} \left(1+\lambda \right)I_{3} & \lambda I_{3}\\ \lambda I_{3} & \left(1+\lambda \right)I_{3} \end{array}\right]$$

provided λ≠−0.5.

Substituting Equation (17) into Equation (16), we get

$$\Rightarrow {\left\|L{\left(I_{6}+\lambda L^{T}L\right)}^{-1}{\hat{p}}_{k}\right\|}_{2}^{2}=\gamma^{2}$$

Substituting Equation (18) and simplifying, we get

$$\Rightarrow \frac{1}{{\left(1+2\lambda \right)}^{2}}{\left\|L{\hat{p}}_{k}\right\|}_{2}^{2}=\gamma^{2}$$

Solving for λ≥0, we get

(19) $$\lambda =0.5\left(\frac{{\left\|L{\hat{p}}_{k}\right\|}_{2}}{\gamma }-1\right)$$

Note that

(20) $${\left\|L{\hat{p}}_{k}\right\|}_{2}={\left\|{\hat{p}}_{k-1}^{S}-{\hat{p}}_{k}^{C}\right\|}_{2}≜{\hat{d}}_{k}$$

Substituting Equation (20) into Equation (19) implies

(21) $$\lambda =0.5\left(\frac{{\hat{d}}_{k}}{\gamma }-1\right)$$

Substituting Equation (18) into Equation (17) and simplifying, we get

(22) $$p_{k}^{C}=\frac{\lambda }{1+2\lambda }{\hat{p}}_{k-1}^{S}+\frac{1+\lambda }{1+2\lambda }{\hat{p}}_{k}^{C}$$

Substituting Equation (21) into Equation (22) implies

(23) $$p_{k}^{C}=\frac{\left({\hat{d}}_{k}-\gamma \right){\hat{p}}_{k-1}^{S}+\left({\hat{d}}_{k}+\gamma \right){\hat{p}}_{k}^{C}}{2{\hat{d}}_{k}}$$

where ${\hat{d}}_{k}$ is the estimated distance of the current foot position relative to the previous stationary period, and $p_{k}^{C}$ is the estimated position of the current time instant.

### 4.2. Applying the Foot Range Constraint Method to the Foot-Mounted ZUPT-Aided PDR System

Based on the analysis of the range constraint algorithm in Section 4.1, it can be applied to the foot-mounted ZUPT-aided PDR system to suppress the positioning drift error during pedestrian movement.

A. Inertial Navigation System

According to the inertial recursion principle, using the IMU raw data (including the three-axis accelerometer and the three-axis gyroscope data), the current navigation state (position, velocity, and attitude angles) can be computed.

(24) $${\hat{x}}_{k}=f\left({\hat{x}}_{k-1},{\tilde{s}}_{k},{\tilde{\omega }}_{k}\right)$$

where ${\hat{x}}_{0}$ is the initial navigation state, ${\hat{x}}_{k}$ is the estimated current navigation state, and ${\tilde{s}}_{k}$ and ${\tilde{\omega }}_{k}$ are the raw data of the three-axis accelerometer and three-axis gyroscope, respectively.

The state-space model ($\delta {\hat{x}}_{k}\in {\mathbb{R}}^{9}$) is described by

(25) $$\delta {\hat{x}}_{k}=F_{k}\delta {\hat{x}}_{k-1}+G_{k}w_{k}$$

where $F_{k}$ and $G_{k}$ denote the state transition and process noise gain matrix, respectively. $w_{k}\in {\mathbb{R}}^{6}$ denotes the perturbation in IMU measurement, which is assumed white and to have the covariance matrix Q. Hence, the state covariance matrix is described by

(26) $$P_{k}=F_{k}P_{k-1}{\left(F_{k}\right)}^{T}+G_{k}Q{\left(G_{k}\right)}^{T}$$

B. Zero-Velocity Update

Using the novel stationary phase detection method in Section 2 to detect the zero-velocity conditions, if the ZUPT is on, then zero-velocity is used as the pseudo-measurement in the Kalman filter framework [7]. The steps involved in applying ZUPT are given below.

(1) Compute the Kalman gain

(27) $$K_{k}=P_{k}{\left(H_{vel}\right)}^{T}{\left[H_{vel}P_{k}{\left(H_{vel}\right)}^{T}+R_{vel}\right]}^{-1}$$

where $H_{vel}=\left[\begin{array}{ccc} 0_{3\times 3} & I_{3\times 3} & 0_{3\times 3} \end{array}\right]$ is the velocity pseudo-measurement observation matrix, and $R_{vel}$ is the velocity pseudo-measurement noise covariance matrix.

(2) Correct the navigation state vector using the velocity pseudo-measurement

(28) $${\hat{x}}_{k}={\hat{x}}_{k}+K_{k}\left[v_{k}-H_{vel}{\hat{x}}_{k}\right]$$

where $v_{k}={\left[\begin{array}{ccc} 0 & 0 & 0 \end{array}\right]}^{T}$ indicates that the velocity is zero.

(3) Correct the state covariance matrix

(29) $$P_{k}=\left[I_{9\times 9}-K_{k}H_{vel}\right]P_{k}$$

C. Range Update

If the distance between the position estimates of the current instance and the previous stationary period is greater than γ (the maximum stride length), then the estimated position by the range constrained algorithm (as explained in Section 4.1) is computed. Using the estimated position as the pseudo-measurement in the Kalman filter framework, the navigation states can be corrected. The steps involved in applying the range update are given below.

(1) Compute the Kalman gain

(30) $$K_{k}=P_{k}{\left(H_{pos}\right)}^{T}{\left[H_{pos}P_{k}{\left(H_{pos}\right)}^{T}+R_{pos}\right]}^{-1}$$

where $H_{pos}=\left[\begin{array}{ccc} I_{3\times 3} & 0_{3\times 3} & 0_{3\times 3} \end{array}\right]$ is the position pseudo-measurement observation matrix, and $R_{pos}$ is the position pseudo-measurement noise covariance matrix.

(2) Correct the navigation state vector using the position pseudo-measurement

(31) $${\hat{x}}_{k}={\hat{x}}_{k}+K_{k}\left[p_{k}-H_{pos}{\hat{x}}_{k}\right]$$

where $p_{k}$ is the range-constrained position estimates.

(3) Correct the state covariance matrix

(32) $$P_{k}=\left[I_{9\times 9}-K_{k}H_{pos}\right]P_{k}$$

The proposed maximum stride-length constraint method is described in Algorithm 3.


**Algorithm 3: The Maximum Stride-Length Constraint Algorithm**
**Initiate:** γ: The maximum stride length, usually 0.8–1.6 m **Input:***zupt*: The detection result of zero-speed points during pedestrian dynamic gait cycles**Output:**$p\in {\mathbb{R}}^{3}$, $v\in {\mathbb{R}}^{3}$, and $\theta \in {\mathbb{R}}^{3}$, the estimated position, velocity, and attitude (roll, pitch, and yaw angles).**Step 1:** Initialize navigation state  Initialize navigation state of the foot-mounted ZUPT-aided PDR system, including initial position, initial heading, and attitude. 
**Step 2:** Zero-velocity update  According to the ZUPT detection result, if the pedestrian is not in the zero-speed interval at the current time instance, use the inertial recursive algorithm to compute the pedestrian’s navigation state; if the pedestrian is in the zero-speed interval, use the ZUPT algorithm to correct the pedestrian’s navigation state of the inertial recursion. **Step 3:** Maximum stride-length update  Compute the foot position distance between the current time instance and the previous stationary instance; if it is greater than the maximum stride length, use the range update algorithm to correct the current pedestrian state (position, velocity, attitude) **Step 4:** Repeat Step 2 and Step 3 until the pedestrian ends the movement.

## 5. Experimental Evaluation

In this section, the three algorithms proposed above are evaluated in a realistic environment. The evaluation results are presented.

### 5.1. Hardware Description

The MTw IMU device from Xsens was used in the evaluation experiment. The IMU included three orthogonally oriented accelerometers, gyroscopes, magnetometers, and one barometer. The data output frequency of MTw was 100 Hz. In the experiment, the IMU device was fixed to the foot to collect pedestrian movement data (see Figure 3). The specification of the MTw device is shown in Table 1.

### 5.2. The Test Routes

#### 5.2.1. Test Route 1

**Figure 14:**
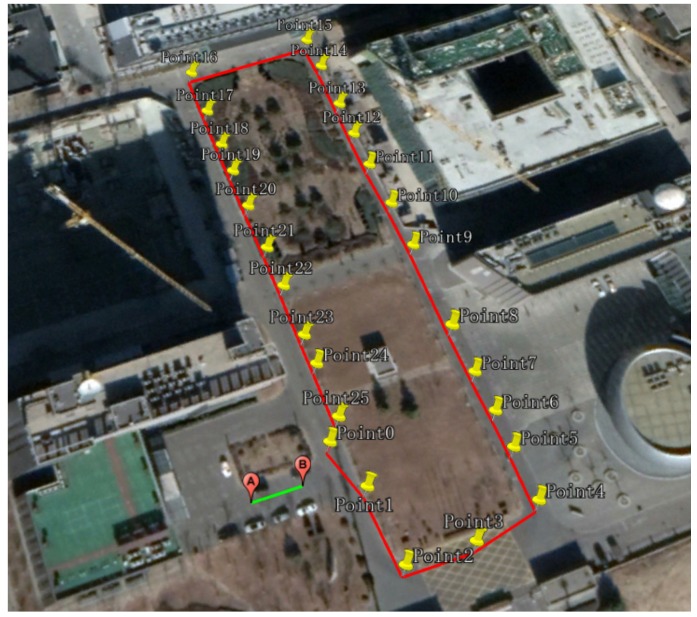
Test route 1.

Test route 1 is shown in Figure 14, which was about 500m. There were 28 reference points with known coordinates (obtained by the DGNSS) laid ahead in the test route. Point A and Point B were the start and end points of the calibration line (the green line in Figure 14) which were used for calibrating the initial heading of the PDR system, and the length of the calibration line was about 12 m. Point 0 was the start point of the test route. Points 0 to 25 formed a reference trajectory (the red line in Figure 14) for evaluating the performance of the foot-mounted PDR system. The experimental data collection process was from Point 0 to 25, then back to Point 0.

#### 5.2.2. Test Route 2

**Figure 15:**
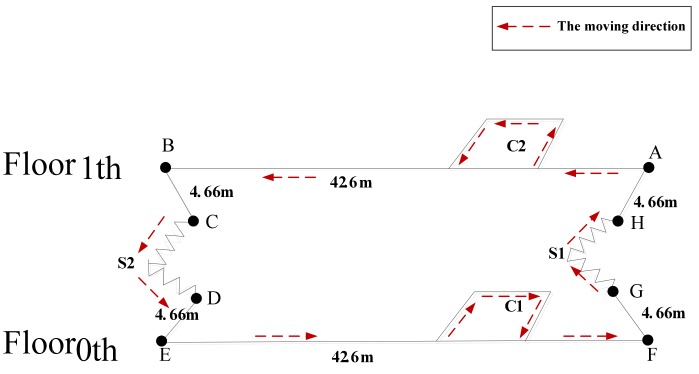
Test route 2.

Figure 15 shows the test route in the building, where C1 and C2 are the spaces beside every floor’s elevator (4.8 m × 7.2 m). S1 and S2 are the stairs between the floors. Point A and Point F represent one side of the corridors, and they coincide with the projection plane, where Point B and Point E are also located. Point C and Point D are the start and end points of the stairs, and they coincide with the projection plane, where Point H and Point G are also located. The experimental route was as follows: starting from Point A, along the corridor, going around in C2, back to the corridor, reaching Point B and taking the stairs S2 down to Floor 0 and reaching Point E, before moving along the corridor, going around in C1, back to the corridor, reaching Point F, then through S1 up to Floor 1, and back to the start point A.

### 5.3. The Initial Absolute Heading Calibrating Experiment

**Figure 16:**
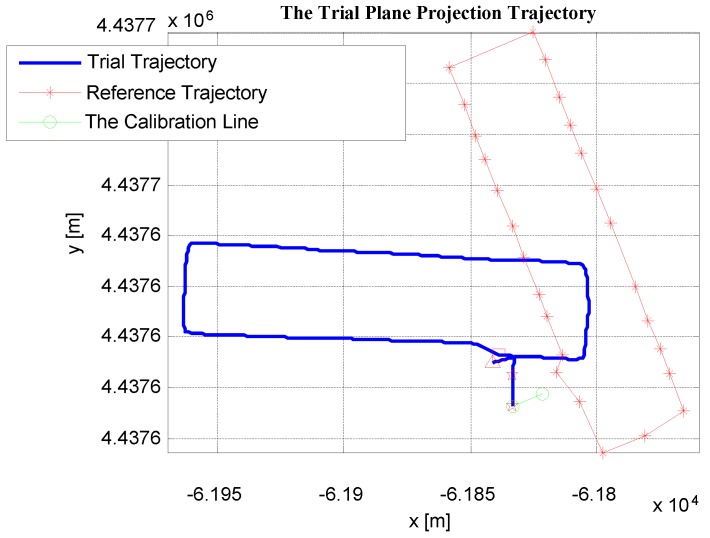
Trial trajectory and reference trajectory in the case of the initial heading being uncalibrated.

As shown in Figure 14, the calibration line consisted of Point A and Point B with known coordinates. The absolute heading $\varphi_{true}$ of the calibration line (from Point A to Point B) could be computed from the coordinate difference of Point A and B, which was $\varphi_{true}=69.1871$ degrees. At the initial stage, the heading of the starting point was set to 0; then, the pedestrian moved along the calibration line (from Point A to Point B). After that, the test trajectory (including the test heading) of this line was obtained by the foot-mounted PDR system, and the test track was directly rotated to coincide with the calibration line according to the angular difference between them. Then, the calibration of the entire test trajectory was completed. After that, in order to evaluate the accuracy of the heading calibration results, the pedestrian started moving from Point 0 and passed every verification point (from Point 1 to Point 25), before heading back to Point 0. By comparing the compliance of the trial trajectory with the reference trajectory, the accuracy of the initial heading calibration results could be evaluated. In Figure 16, the trial trajectory and the reference trajectory in the case of the initial heading being uncalibrated are shown.

#### 5.3.1. Using the Proposed Method to Calibrate the Initial Heading of the PDR System

**Figure 17:**
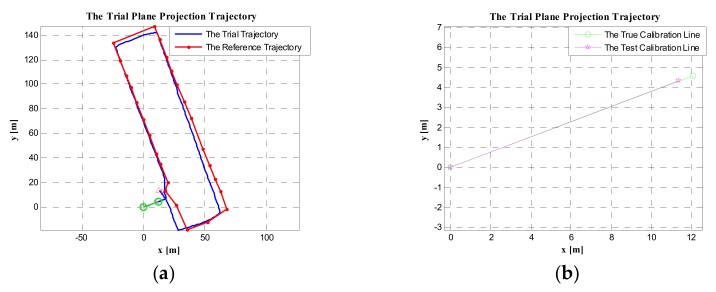
The trial plane projection trajectory. (**a**) The trial trajectory and reference trajectory in the case of the initial heading being calibrated using a known calibration line. (**b**) The coincidence of the test calibration line (after rotating) with the true calibration line.

Using the test coordinate difference of the start and end points of the calibration line, the test heading of the calibration line could be obtained, which was denoted as $\varphi_{test}$. In this experiment, by computation, $\varphi_{test}=1.7969$ degrees. Hence, the initial heading bias of the PDR system relative to the true heading was $\Delta \varphi =\varphi_{true}-\varphi_{test}=69.1871-1.7969=67.3902$ degrees. Then, the initial heading of the trial trajectory was calibrated by rotating 67.3902 degrees. The trial trajectory after rotating and the reference trajectory are shown in Figure 17a. The test calibration line (after rotating) and the true calibration line are shown in Figure 17b. As can be seen from Figure 17, the trial trajectory after calibration and the reference trajectory had good coincidence.

**Figure 18:**
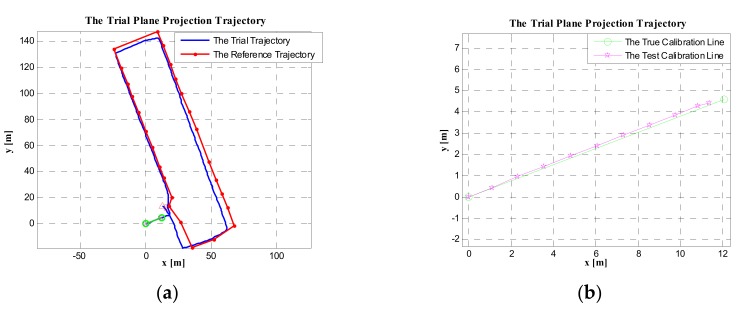
The trial plane projection trajectory. (**a**) The trial trajectory and reference trajectory in the case of the initial heading being calibrated using a known calibration line (averaging the heading between each pair of adjacent points). (**b**) The coincidence of the test calibration line (after rotating) with the true calibration line.

As described in the Section 2, the test heading value of the calibration line can also be obtained by averaging the heading between adjacent points. When the pedestrian walks along the calibration line, all the test coordinates of the calibration line (each time the foot touches the ground) can be obtained from the PDR system. by averaging all the headings between each pair of adjacent points, the mean test heading can be obtained, denoted as $\varphi_{ave}$. In this experiment, $\varphi_{ave}=2.1796$ degrees. Therefore, the heading bias of the test PDR system relative to the true heading (the true heading of the calibration line) was $\Delta \varphi =\varphi_{true}-\varphi_{ave}=69.1871-2.1796=67.2275$ degrees. Then, rotating the test trajectory with the heading bias angle of 67.0075 degrees, the initial heading of the test trajectory was calibrated. The trial trajectory after rotating and the reference trajectory are shown in Figure 18a. The test calibration line (after rotating) and the true calibration line are shown in Figure 18b. By comparing Figure 17 and Figure 18, we found that the coincidence of the trial trajectory after calibration with the reference trajectory was almost same. However, the coincidence of the test calibration line (after rotating) and the true calibration line in Figure 17b was better than that in Figure 18b. The reason is that $\varphi_{ave}$ may have averaged some deviation headings (see Figure 2). In other words, the initial heading computed only using the coordinate difference of the start and end points was more representative of the true heading of the calibration line.

#### 5.3.2. Using the Magnetic Heading of the Start Point to Calibrate the Initial Heading of the PDR System

**Figure 19:**
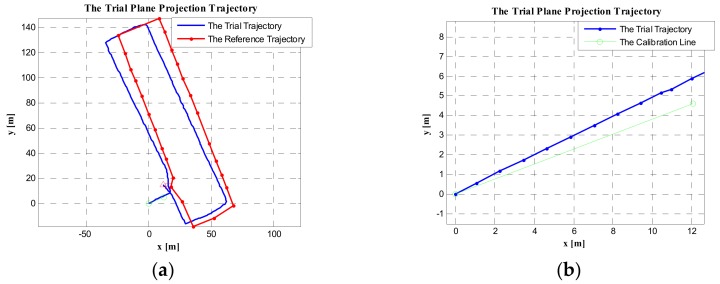
The trial plane projection trajectory. (**a**) The trial trajectory and reference trajectory in the case of the initial heading being calibrated using the magnetic heading of the start point. (**b**) The coincidence of the trial trajectory (after calibration) with the true calibration line.

Using the three-axis magnetic field strength of the start point (Point A), the magnetic heading could be obtained, which was 69.1551 degrees, and the local magnetic declination of the Beijing experiment site was −6.95 degrees; thus, the absolute heading of the start point was 62.2051 degrees. Using this heading to calibrate the trial trajectory, the result is shown in Figure 19a, compared with the reference trajectory. In Figure 19b, the coincidence of the trial trajectory (after calibration) with the calibration line is shown. As we can see, in Figure 19, the trial trajectory deviated from the reference trajectory. Therefore, the magnetic heading could not effectively calibrate the initial heading of the test trajectory.

#### 5.3.3. Directly Using the Geographical Heading of the Start Point to Initialize the Heading of the PDR System

**Figure 20:**
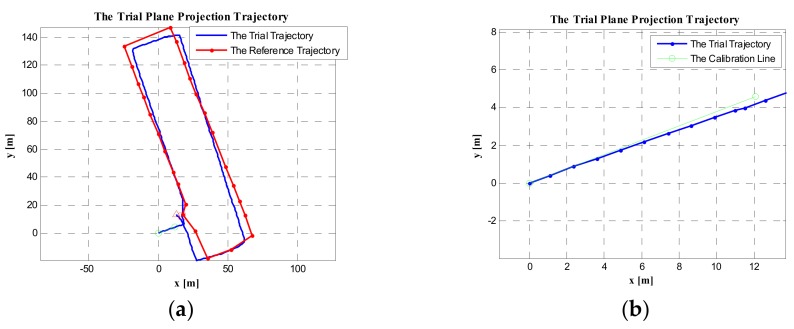
The trial plane projection trajectory. (**a**) The trial trajectory and reference trajectory in the case of the initial heading being calibrated by using the absolute geographical heading of the start point. (**b**) The coincidence of the trial trajectory (after calibration) with the true trajectory of the calibration line.

The heading of the start point (Point A) was the same as the heading of the calibration line (AB), that is, $\varphi_{start}=\varphi_{true}=69.1871$ degrees. When directly using $\varphi_{start}$ as the initial heading of PDR system, the result was as shown in Figure 20. Compared with the trial trajectories in Figure 17 and Figure 18, the trial trajectory in Figure 20a more obviously deviated from the reference trajectory. In Figure 20b, the coincidence of the trial trajectory (after calibration) with the true calibration line is shown. Similarly, the trial trajectory in Figure 20b more obviously deviated from the true trajectory than that in Figure 17 and Figure 18. Therefore, directly using the absolute heading of the start point is less effective than the method (using the calibration line) we proposed.

By comparing the calibration results of the above four methods, the method using the calibration line was more effective than using the geomagnetic heading and the geographical heading of the starting point. Meanwhile, by comparing Figure 17 and Figure 18, they mainly had the same effect when calibrating the initial heading of the PDR system. However, in Figure 17b, the coincidence of the test calibration line (relative to the true calibration line) was better than that in Figure 18b. Therefore, the method of only using the coordinate difference of the start and end points to compute the initial heading of the calibration line was the most effective method to calibrate the initial heading of the PDR system.

### 5.4. The Stationary Phase Detection Experiment

Since experimental route 2 included stairs, pedestrians could go up and down stairs and perform various types of movements in the corridor, effectively evaluating the effectiveness of the zero-speed interval detection algorithm proposed in this paper. Therefore, we used experimental route 2 to verify the zero-speed interval detection algorithm proposed in this paper. At the same time, as a comparison, the same test data were processed by the GLRT algorithm, and the obtained pedestrian positioning trajectory using the GLRT algorithm was compared with the trajectory processed by the proposed algorithm.

The experimental data collection process was as follows: the pedestrian started movement from point A of Floor 1, walked along the corridor, went around in C2, then ran along the corridor for a while, before reaching point B, going down the stairs from S2, and arriving at point E of Floor 0. On Floor 0, similarly to Floor 1, the pedestrian walked along the corridor, then ran for a while, before going around in C1, going upstairs through S1, and finally arriving at the starting point A of Floor1.

#### 5.4.1. Test Using the GLRT Method

**Figure 21:**
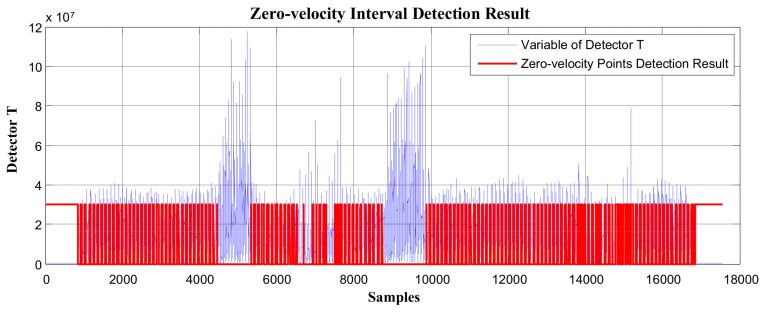
The zero-velocity interval detection result using the GLRT method.

**Figure 22:**
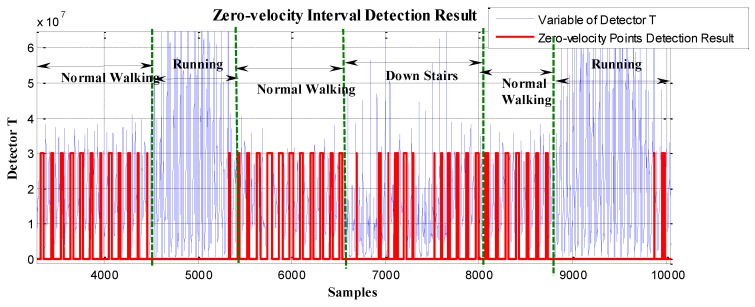
The zero-velocity interval detection result using the GLRT method.

Using the GLRT method to process the test data collected in experimental route 2, the overall zero-speed interval detection result was as shown in Figure 21. In Figure 21, the horizontal axis represents the sampling points, and the vertical axis is the variable of detector *T*. The red line in the figure represents the stationary state and moving state, where large values indicate the stationary interval, while small values indicate the moving interval. As we can see, between Sample 4000 and Sample 5000, there were obviously missing detections. Similarly, there were also missing detections between Sample 8000 and Sample 10,000. In order to better observe the missing detections, the detection results from Sample 4000 to Sample 10,000 are enlarged and displayed in Figure 22.

In Figure 22, as we can see, the GLRT method performed well during normal walking, but when the pedestrian was in the running state (around Sample 5000), it failed to detect the zero-velocity interval and had missing detections during this period. Similarly, after switching from walking to going downstairs (around Sample 7000), there were obviously failed detections during this period.

**Figure 23:**
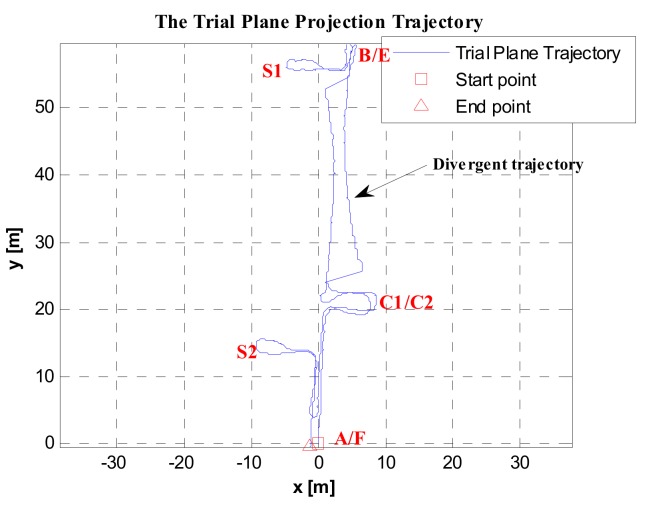
Pedestrian positioning trajectory based on the GLRT method.

Based on the zero-speed interval detected results shown in Figure 21 and Figure 22, the pedestrian plane positioning trajectory is shown in Figure 23. The trajectory from C1/C2 to the side of point B/E should be a straight line (see Figure 15), and the trajectories of Floor 0 and Floor 1 should be basically coincident. However, in Figure 23, since the GLRT method obviously failed to detect during the running state and going downstairs, there was a serious shift in the pedestrian positioning trajectory.

#### 5.4.2. Test Results Using the Proposed Method

**Figure 24:**
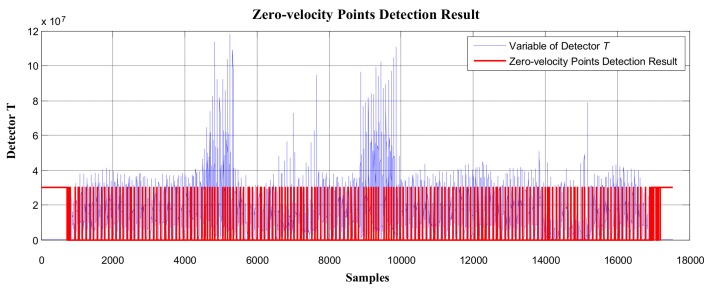
The zero-velocity interval detection result using the proposed method.

**Figure 25:**
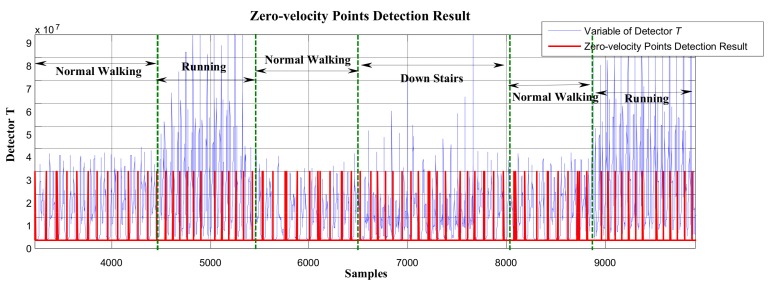
The zero-velocity interval detection result using the proposed method.

As stated in Section 3.3, regardless of the type of motion (including walking, running, stair-climbing, going downstairs, etc.), generally, at least one gait cycle can be completed within 0.7–1.2 s. In the experiment, using the proposed method to process the same test data collected in experimental route 2, the minimum periodic time window (gait-cycle duration) was chosen as 1 s to divide the pedestrian movement into independent gait cycles. The sampling frequency of the IMU was 100 Hz, which means that, for every 100 data points collected, the zero-speed point detection was performed. The overall zero-speed point detection results are shown in Figure 24. Compared to the missing detections in Figure 21, the proposed method effectively detected the zero-speed points for each periodic time window as shown in Figure 24. The partial enlargement result of Figure 24 is also shown in Figure 25.

In Figure 24 and Figure 25, the red line represents the stationary state and moving state, where large values indicate the stationary interval, and small values indicate the moving interval. Similarly, compared to the missing detections in Figure 22, the proposed method performed well (see Figure 25) not only during normal walking, but also during the running state and going downstairs.

**Figure 26:**
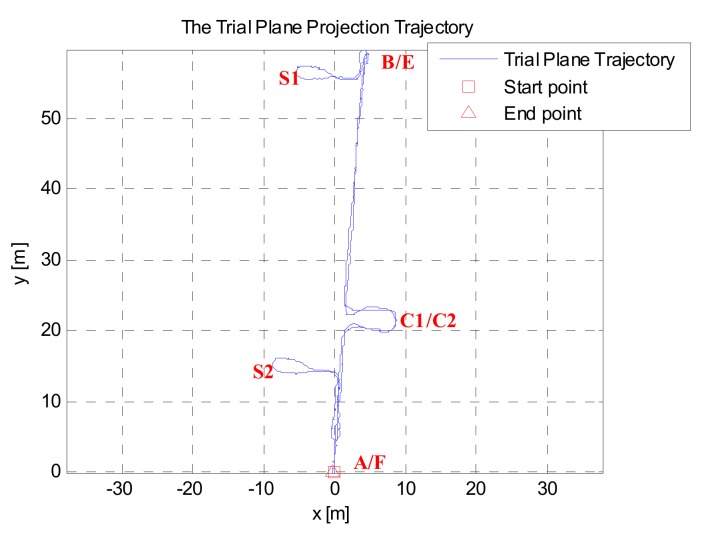
Pedestrian positioning test trajectory processed using the proposed method.

Based on the zero-speed interval detection results using the proposed method (as shown in Figure 24 and Figure 25), the pedestrian plane positioning trajectory is shown in Figure 26. Compared to the offset and divergence of the trajectory in Figure 23, the positioning result in Figure 26 shows considerable improvement. The obtained trajectory is basically the same as the experimental route, and the trajectories of Floor 0 and Floor 1 are coincident.

In addition, when using the proposed method to detect the zero-speed points, only the minimum value point of detector *T* was detected as the zero-speed point of each periodic cycle (see Figure 24 and Figure 25). As we can see, the pedestrian positioning result (see Figure 26) is basically the same as the true route. The total length of the experimental route was about 259.3819 m, but the closing error was only 0.2085 m. Thus, it is verified that, as long as one zero-speed point is detected correctly in each periodic cycle, the positioning errors of the inertial recursive results can be effectively suppressed.

### 5.5. The Maximum Stride-Length Constraint Algorithm Experiment

In the long-distance experimental scenario, the heading of the foot-mounted ZUPT-aided PDR system is more prone to divergence over time. Since experimental route 1 was about 500 m in length, it was selected to verify the effectiveness of the maximum stride-length constraint algorithm.

The experimental data collection process was as follows: the pedestrian started walking from point A (see Figure 14), then walked following the direction of Point 1→Point 2→…→Point 25→Point 0→Point 1, passing every reference point; then, the pedestrian took another lap along the same path, back to Point A. The total length of the experimental route was about 1.1588 km.

The maximum stride length γ was obtained by the actual measurement of the stride length of the volunteer participated in the experiment, and it was around 1.15 m. Then, by comparing the compliance of the trial trajectory with the reference trajectory (by converting verification points into lines) and the closing error of the trial trajectory, the effectiveness of the maximum stride-length constraint algorithm was verified.

**Figure 27:**
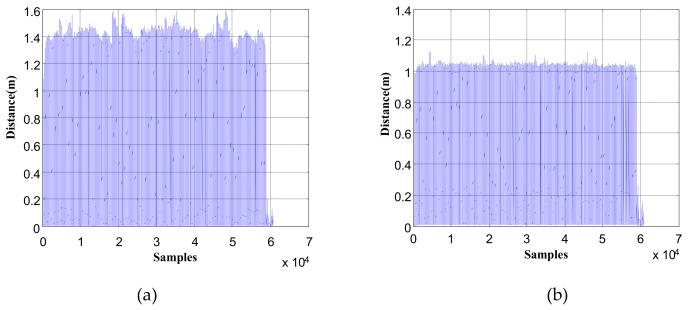
The distance between the current foot position and the foot position of the previous stationary period: (**a**) without maximum stride-length constraint; (**b**) with maximum stride-length constraint.

In Figure 27, the distance between the current foot position (all time instances) and the foot position of the previous stationary period (relative to the current time instance) is shown. Figure 27a is the distance result without using the stride-length constraint algorithm, where the distance range was 0–1.6 m, However, the maximum stride length of the pedestrian involved in the experiment was about 1.15 m; thus there were lots of distances in Figure 27a beyond the maximum stride length. In Figure 27b, the proposed maximum stride-length constraint algorithm was used, and the maximum stride length was set to 1.15 m; as can be seen, the distance ranges of all time instances became 0–1.2 m.

**Figure 28:**
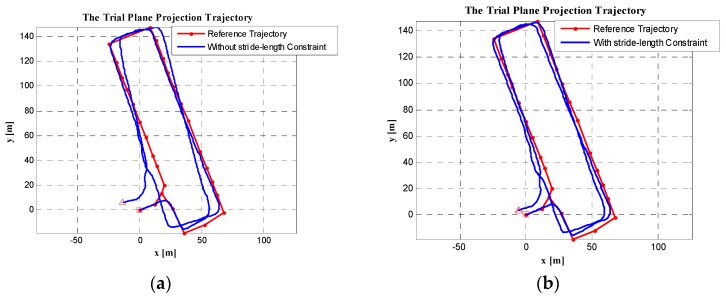
The test pedestrian trajectories: (**a**) without maximum stride-length constraint; (**b**) with maximum stride-length constraint.

The trial trajectory is shown in Figure 28, where the red line is the reference trajectory consisting of the ground reference points, and the blue line is the trial trajectory. In Figure 28a, the trial trajectory was obtained using the ZUPT-aided PDR system. As can be seen, the experimental trajectory clearly deviated from the reference trajectory, especially on the second lap. In Figure 28b, the trial trajectory was obtained not only using the ZUPT-aided PDR system, but also using the maximum stride-length constraint algorithm; here, the trial trajectory deviation from the reference trajectory showed considerable improvement. The first and second laps of the experimental trajectory were substantially coincident with the reference trajectory. In particular, the red square and triangle (the start and the end points of the trial trajectory) almost overlapped.

In Table 2, we list the trial trajectory‘s closure error before and after using the maximum stride-length constraint algorithm. It can be seen from the table that, when the algorithm was not used, the trajectory plane closure error was 15.199 m, and the closure error divided by the trajectory length was 1.3%. However, with this algorithm, the trajectory plane closure error was reduced to 6.6062 m, and the closure error divided by the trajectory length was reduced to 0.57%. Therefore, the algorithm effectively constrained the positioning error of the trajectory.

## 6. Conclusions

In this paper, a new method to calibrate the PDR system’s initial absolute heading was proposed. By using a calibration line rather than only using the heading of the starting point, the method could calibrate the initial heading more accurately. Then, a novel stationary phase detection method was proposed based on foot motion periodicity rather than the threshold comparison principle in the traditional method. Through an experiment, we found that the zero-speed state points always occurred around the minimum value of stationary detector *T* in each gait cycle. By taking the minimum value in each gait cycle as the zero-speed state point, we could effectively reduce the failed detection of zero-speed points. At last, a new motion constraint method was exploited, based on the range constraint principle. During the pedestrian movement, the distance between the foot position estimates of the current moment and the previous stationary period was within the maximum stride length. Once the distance was greater than the maximum stride length, the constraint algorithm was used to confine the current estimated foot position to the sphere of the maximum stride length relative to the previous stationary foot position.

In the maximum stride-length constraint algorithm, the maximum stride length we used was a fixed value. Actually, the maximum stride length of each step changes with the gait. Therefore, the maximum stride length should not be fixed; this will be addressed and refined in future work.

## Figures and Tables

**Table 1:** The specification of the MTw inertial measurement unit (IMU) device. ACC—accelerometer; GYR—gyroscope; MAG—magnetometer; BAR—barometer.

Algorithm Type	ACC	GYR	MAG	BAR
Sensor type	Analog	Analog	Digital	Digital
Full scale	±160 m/s^2^	±1200 deg/s	±1.5 Gauss	300–1100 hPa
Linearity	0.2%	0.1%	0.2%	0.05%
Bias stability	-	20 deg/hour	-	100 Pa/year
Noise	$0.003\;m/s^{2}/\sqrt{\mathrm{Hz}}$	$0.05\;\deg/s/\sqrt{\mathrm{Hz}}$	$0.15\;m\mathrm{Gauss}/\sqrt{\mathrm{Hz}}$	$0.85\;\mathrm{Pa}/\sqrt{\mathrm{Hz}}$

**Table 2:** The assessment results of the two algorithms.

Algorithm Type	Closure Error (m)	Closure Error/Length
Without stride-length constraint	15.199	1.3%
With stride-length constraint	6.6062	0.57%
